# Mitochondrial peptide MOTS‐c suppresses systemic and cardiac inflammasome activation in a diabetic rat model

**DOI:** 10.1113/EP093714

**Published:** 2026-06-19

**Authors:** Aimee R. Mills, Antonio de Souza, Toan Pham, Odunayo O. Mugisho

**Affiliations:** ^1^ Buchanan Ocular Therapeutics Unit Department of Ophthalmology, New Zealand National Eye Centre University of Auckland Auckland New Zealand; ^2^ Centre for Brain Research University of Auckland Auckland New Zealand; ^3^ Auckland Bioengineering Institute University of Auckland Auckland New Zealand; ^4^ Department of Engineering Science and Biomedical Engineering University of Auckland Auckland New Zealand

**Keywords:** diabetic heart, inflammasome, inflammation, MOTS‐c

## Abstract

Type 2 diabetes mellitus (T2DM) is associated with chronic systemic and cardiac inflammation, contributing to the development of diabetic cardiomyopathy. The mitochondrial‐derived peptide mitochondrial open reading frame of the 12S rRNA type‐C (MOTS‐c) has emerged as a promising regulator of metabolic and inflammatory pathways. In this study, we investigated the effects of MOTS‐c treatment on leucine‐rich repeat and pyrin domain‐containing receptor 3 (NLRP3) inflammasome activity in a high‐fat diet and streptozotocin‐induced T2DM rat model. MOTS‐c treatment significantly reduced fasting blood glucose and circulating C‐reactive protein levels, while selectively modulating plasma inflammatory cytokines, including interleukin (IL)‐10 and IL‐1β. Immunohistochemical analysis revealed reduced levels of NLRP3, apoptosis‐associated speck‐like protein containing a caspase activation and recruitment domain (ASC), and cleaved caspase‐1 in left ventricular tissue following MOTS‐c administration. Correlation analyses linked IL‐18 and IL‐1β with elevated markers, including low‐density lipoprotein and uric acid, suggesting interplay between overall health and inflammasome activity. These results indicate that MOTS‐c modulates both systemic and cardiac inflammation in T2DM, providing a novel therapeutic approach for reducing cardiovascular risk in diabetic patients.

## Abstract (highlights)

- **What is the central question of this study?**
  How does MOTS‐c treatment affect cardiac inflammation pathways and systemic inflammation in Type 2 diabetes?
- **What is the main finding and its importance?**
  MOTS‐c treatment lowered fasting blood glucose levels, circulating C‐reactive protein and key inflammatory cytokines in type 2 diabetic rats, while reducing NLRP3 and cleaved caspase‐1 levels in the heart. Correlations between IL‐18 and IL‐1β, low‐density lipoprotein and uric acid highlight the links between metabolic dysfunction and inflammation. Our findings suggest that MOTS‐c suppresses systemic and cardiac inflammation pathways in diabetes, highlighting its potential to reduce cardiovascular risk.

## 1 INTRODUCTION

Type 2 diabetes mellitus (T2DM) is a common chronic disease characterised by persistently high blood glucose levels. T2DM is associated with a range of microvascular and macrovascular complications, which significantly increase the risk of dysfunction across multiple organ systems. Cardiovascular complications remain the leading cause of morbidity and mortality in T2DM patients. Alongside impaired vasculature, T2DM also directly affects the myocardium with altered substrate metabolism and left ventricular hypertrophy, leading to decreased contractile function and heart failure, which accounts for more than 80% of all deaths (Boudina & Abel, 2007). A better understanding of the underlying processes of T2DM‐induced heart dysfunction is crucial for establishing effective therapeutic options, yet this remains an ongoing challenge for healthcare and biomedical research.

The heart is a high‐energy‐demanding organ and requires a continuous supply of ATP to support constant mechanical work. Mitochondria primarily supply ATP via oxidative phosphorylation and occupy approximately 35% of the total volume of mammalian cardiac myocytes (Barth et al., 1992). Beyond their fundamental role in generating ATP, mitochondria are also the primary source of reactive oxygen species (ROS). In T2DM, hyperglycaemia exacerbates excessive mitochondrial ROS production, triggering oxidative damage, inducing mitochondrial dysfunction and promoting higher inflammation (Kaneto et al., 2010). Emerging evidence has reported cardiac mitochondrial structural abnormalities (Tang et al., 2023) and decreased bioenergetic function in both T2DM patients and animal models (Croston et al., 2014; Pham et al., 2025).

Alongside mitochondrial dysfunction, chronic elevation of inflammation is a contributing factor in the pathogenesis of T2DM. Chronic inflammation has been reported in patients with T2DM and in animal models (Jiang et al., 2022; Tschöpe et al., 2005). Specifically, the leucine‐rich repeat and pyrin domain‐containing receptor 3 (NLRP3) inflammasome appears to be active in T2DM models and linked to cardiac remodelling and dysfunction (Luo et al., 2014). Numerous inflammatory cytokines, including interleukin (IL)‐6, tumour necrosis factor α (TNF‐α), and the inflammasome‐derived IL‐1β and IL‐18, have been found to be elevated in the systemic circulation of T2DM patients (Mirza et al., 2012; Rodrigues et al., 2017; Suchanek et al., 2005) and in heart tissues of diabetic models (Luo et al., 2014; Maedler et al., 2002). These chronic elevations perpetuate further tissue remodelling and excessive ROS production, which in turn contribute to the progression of cardiac mitochondrial dysfunction and ultimately heart failure (Bellemare et al., 2025; Luo et al., 2014; Maedler et al., 2002).

Mitochondrial open reading frame of the 12S rRNA type‐C (MOTS‐c) is a newly discovered mitochondria‐derived peptide encoded by the open reading frame in mitochondrial 12S rRNA. This small 16‐amino‐acid protein is highly conserved among species (Lee et al., 2015). Emerging evidence shows that MOTS‐c plays a key role in regulating cellular metabolism by enhancing glucose utilisation and stress response through the activated AMP‐activated protein kinase (AMPK) pathway (Fang et al., 2025; Lee et al., 2015; Wu et al., 2025). Clinical and experimental studies have reported lower blood MOTS‐c levels in T2DM individuals (Ramanjaneya et al., 2019), gestational diabetes (Yin et al., 2022) and diabetic mice (Xu et al., 2024). Therapeutic MOTS‐c administration has shown multiple benefits, including improved heart function in diabetic rats by enhancing glucose metabolism (Li et al., 2022), mitochondrial function (Pham et al., 2025) and antioxidant defences (Tang et al., 2023). MOTS‐c has also been shown to prevent insulin resistance in high‐fat‐diet‐induced obesity (Lee et al., 2015) and attenuate cardiac hypertrophy induced by pressure overload (Zhong et al., 2022) and T2DM (Pham et al., 2025).

The anti‐inflammatory properties of MOTS‐c have been reported in several disease conditions (Jiang et al., 2023; Wu et al., 2023, 2025). A protective effect of MOTS‐c against diabetic cardiomyopathy has been reported in a mouse model of type 1 diabetes (T1D) by activating the AMPK pathway and inhibiting inflammation in heart tissues (Wu et al., 2025). MOTS‐c attenuated myocardial inflammation in lipopolysaccharide‐induced septic cardiomyopathy mice by decreasing mRNA levels of IL‐1β, IL‐4, IL‐6 and TNFα, while decreasing serum levels of markers of myocardial injury (creatine kinase‐MB, troponin T and TNF‐α) improved myocardial dysfunction (Wu et al., 2023). Treatment with MOTS‐c (0.5 mg/kg/day) for 8 weeks reduced the expression of ROS/thioredoxin‐interacting protein/NLRP3 pathway proteins, thereby inhibiting the diabetic myocardial inflammatory response (Fu et al., 2024). These findings suggest that MOTS‐c may have a protective effect by interfering with inflammatory pathways that contribute directly to cardiac damage. However, the direct effect of MOTS‐c on cardiac inflammation pathways and systemic inflammation in T2DM remains unknown.

We hypothesise that MOTS‐c therapy may alleviate inflammation and improve general health in T2DM by reducing NLRP3 inflammasome activity. This study examined how MOTS‐c treatment altered inflammatory responses in a well‐established T2DM rat model. This rat model closely resembles the metabolic abnormalities of the heart observed in diabetic patients (Mansor et al., 2013).

## 2 METHODS

### 2.1 Ethical approval

All animal handling procedures were conducted in accordance with protocols approved by the University of Auckland Animal Ethics Committee (AEC 22653) and followed the guidelines from Directive 2010/63/EU of the European Parliament on the protection of animals used for scientific purposes. Male Wistar rats (6–7 weeks old, 150–200 g) were purchased from the animal facility (Vernon Jansen Unit) at The University of Auckland. The rats were randomly assigned to three groups: control (*n* = 12), untreated diabetic (*n* = 7) and treated diabetic (*n* = 11). Diabetes was induced using a combination of dietary and pharmacological interventions. Rats were fed with a high‐fat diet (HFD, 43% digestible energy from lipids, SF04‐001, Specialty Feeds, Glen Forrest WA, Australia) for a total duration of 15 weeks and received a low‐dose intraperitoneal injection of streptozotocin (STZ, 25 mg/kg, in citrate buffer pH 4) at week 8 post‐HFD feeding. Control rats received standard chow and an injection of citrate buffer. At week 12, the treated diabetic group received daily intraperitoneal injections of MOTS‐c (15 mg/kg/day) for 3 weeks, while the untreated diabetic and control groups received saline injections. The MOTS‐c dose was based on prior studies demonstrating efficiency in metabolic effects, which used higher doses for shorter durations (Reynolds et al., 2021). All rats had access to water and food in a 12‐h light–dark cycle. Body weight and blood glucose were monitored weekly. At week 15, each rat was fasted for 6 h and then received an intraperitoneal glucose bolus (2 g/kg in sterile saline) for a glucose tolerance test. Blood samples were then obtained from the tail puncture. Blood glucose was measured using an Accu‐Chek (Indianapolis, Indiana, USA) blood glucose meter at various time points (before the glucose tolerance test, then 15, 30, 60, 90 and 120 min after glucose administration). We excluded some rats with Type 1 diabetic signs, including hyperglycaemia (>30 mmol/L), polyuria and substantial weight loss. Therefore, group sizes were unequal across the three groups.

### 2.2 Drug

Mitochondria‐derived peptide MOTS‐c (MRWQEMGYIFYPRKLR) was synthesised by GenScript Biotech Ltd (Singapore) and kept in powder form at −80°C. MOTS‐c was freshly dissolved in sterile saline before injection and used within 30 min.

### 2.3 Heart tissue collection and fixation

On the experiment day, each rat was transported to our department (Auckland Bioengineering Institute within the University of Auckland) and placed in a climate‐controlled chamber with access to food and water for at least 1 h to minimise any stress arising from transportation. Rats were deeply anaesthetised with isoflurane inhalation (5% in O_2_) within 10 min and subcutaneously injected with heparin (1000 IU/kg). Following cervical dislocation, the heart was dissected, submerged in cold Tyrode solution, and then Langendorff‐perfused with oxygenated Tyrode solution at room temperature. The Tyrode solution contained (in mmol/L): 130 NaCl, 6 KCl, 1 MgCl_2_, 0.3 CaCl_2_, 0.5 NaH_2_PO_4_, 10 HEPES, 10 glucose, and 20 2,3‐butanedione monoxime (pH 7.4, adjusted with Tris). Heart mass and tibial length were measured.

Small blocks of left ventricular tissues were fixed with 2% paraformaldehyde in phosphate‐buffered saline (PBS) for 1 h at 4°C before being cryoprotected with 30% sucrose in PBS. The tissue blocks were placed in Tissue‐Tek OCT buffer and stored frozen at −80°C for later cryosectioning and immunolabeling.

### 2.4 Fluorescence immunohistochemistry

Six left ventricular blocks per group were selected at random and sectioned at 14 µm using a cryostat for fluorescence immunohistochemistry (fIHC). Sections were washed with PBS 3 times for 5 min each, blocked with Immunobuffer consisting of PBS with 0.1% Triton X‐100 and 10% normal horse serum for 1 h at room temperature, before 24 h incubation at 4°C with the following primary antibodies diluted in Immunobuffer: NLRP3 (1:200, Abcam, Waltham, MA, USA, cat. no. ab4207, RRID: AB_955792), Apoptosis‐associated Speck‐like protein containing a Caspase activation and recruitment domain (ASC, 1:200, AdipoGen, San Diego, CA, cat. no. AG‐25B‐0006, RRID: AB_2490440), and cleaved caspase‐1 (CC1, 1:100, Thermo Fisher Scientific, Waltham, MA, USA, cat. no. PA5‐38099, RRID: AB_2554702). Sections were washed 3 × 5 min in PBS before 2 h incubation at room temperature with 4′,6‐diamidino‐2‐phenylindole (DAPI, 1:2000) and the following secondary antibodies diluted in PBS: donkey anti‐goat Cy3 (1:500, Jackson ImmunoResearch Laboratories, West Grove, PA, USA, cat. no. 705‐165‐147, RRID: AB_2307351) and donkey anti‐rabbit Cy5 (1:500, Jackson ImmunoResearch, cat. no. 711‐175‐152, RRID: AB_2340607). Sections were washed three times for 5 min, covered with Citifluor (Electron Microscopy Sciences, Morgantown, PA), and imaged using the FV1000 confocal laser scanning microscope (Olympus, UPlanSApo ×60 oil objective). Nine images were acquired per group by a masked researcher, and fluorescence signal was quantified for each marker as percentage area coverage after applying a set threshold to all images using ImageJ 1.54p (W.S. Rasband, National Institutes of Health, Bethesda, MD, USA).

### 2.5 Blood plasma collection and analysis

Blood samples were also collected immediately after cervical dislocation and heart excision by drawing remaining blood from the opened thoracic cavity using a sterile syringe and transferring it into a 5 mL EDTA‐vacuum blood tube. The plasma sample was then separated by centrifugation at 2000 *g* for 10 min at 4°C and stored in a −80°C freezer for later analysis. Metabolite concentrations were measured on a Hitachi c311 autoanalyser (Hitachi High Technologies Corporation, Tokyo, Japan). Plasma concentrations of glucose, urea, alanine aminotransferase (ALT), albumin, lactate dehydrogenase (LDH), aspartate aminotransferase (AST), creatinine, uric acid, and lipid profile markers (high‐density lipoprotein (HDL), low‐density lipoprotein (LDL) and Triglycerides) were measured using colorimetric assays. C‐reactive protein (CRP) was analysed by particle‐enhanced immunoturbidimetric assay (Roche Diagnostics, Mannheim, Germany).

### 2.6 Luminex cytokine assay

Blood plasma samples were assessed using a rat Magnetic Luminex® Discovery Assay (Bio‐Techne, Minneapolis, MN, cat. no. LKSAHM‐16) to quantify the following cytokines: IL‐1β, IL‐18, IL‐10, IL‐6, TNF‐α, interferon γ (IFN‐γ) and intercellular adhesion molecule‐1 (ICAM‐1). Blood samples, run in triplicate, were diluted 1:2 using RD2‐1. Magnetic beads pre‐coated with capture antibodies were added with samples, standards and blanks in a 96‐well plate to initiate analyte binding. After washing, a biotinylated detection antibody was added, followed by streptavidin–phycoerythrin conjugate. A final wash was performed before using a Luminex MAGPIX System to measure the intensity of selected analytes.

### 2.7 Statistical analysis

Outliers were identified and removed using ROUT outlier analysis (*Q* = 2%). The Shapiro–Wilk and Brown–Forsythe tests were conducted to assess normality and homogeneity of variances, respectively. As all data showed normal distributions and equal variances, analysis was performed using an ordinary one‐way ANOVA with Dunnett's multiple‐comparison test. Otherwise, analysis was performed using the Brown–Forsythe ANOVA with Dunnett's T3 multiple‐comparison test. Pearson correlation analysis was used to characterise the relationships between circulating markers and inflammasome cytokines: IL‐1β and IL‐18. Pearson correlation coefficient (*r*) values used to describe these relationships were defined as follows: very weak (±0 to ±0.19), weak (±0.2 to ±0.39), moderate (±0.40 to ±0.59), strong (±0.6 to ±0.79) and very strong (±0.8 to ±1). All data are presented as means ± SD. A significant difference was considered when *P* < 0.05. Prism software was used to generate graphs (GraphPad Software, Boston, MA, USA).

## 3 RESULTS

**FIGURE 1:**
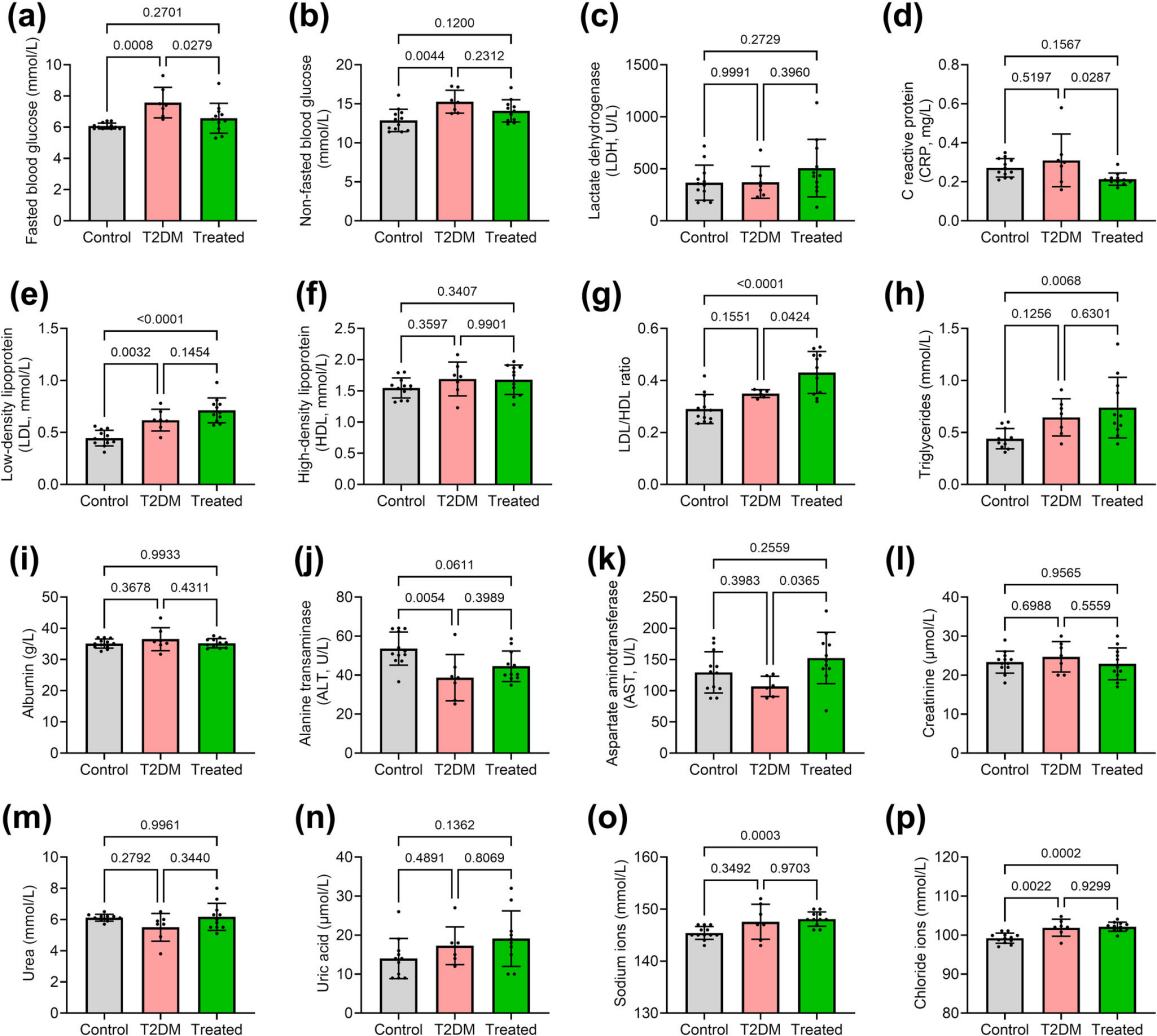
Circulating markers of general health in the T2DM rat model and effect of MOTS‐c treatment. Circulating markers of metabolism and inflammation (green), general cardiac health (orange), liver function (purple) and kidney function (blue) in control rats, type 2 diabetes mellitus (T2DM) rat, and T2DM rats treated with mitochondrial open reading frame of the 12S rRNA type‐C (MOTS‐c). Data are means ± SD. Control (*n* = 12), T2DM (*n* = 7), Treated (*n* = 11). Ordinary one‐way ANOVA with Dunnett's multiple comparison of all groups to the T2DM group; Brown–Forsythe ANOVA with Dunnett's T3 multiple comparison of all groups to the T2DM group.

Morphological characteristics of three rat groups and glucose tolerance tests have been previously reported (Pham et al., 2025). Briefly, untreated T2DM rats showed increased body mass, tibial length, heart mass, left ventricular thickness and blood glucose concentration compared with controls. MOTS‐c treatment alleviated these increases in T2DM rats. Blood samples were analysed for circulating metabolic markers linked to metabolism, inflammation, cardiac health, liver function and kidney function (Figure 1). In T2DM rats, fasted blood glucose (*P *= 0.0008; Figure 1a) and non‐fasted blood glucose (*P* = 0.0044; Figure 1b) were significantly higher compared to controls. MOTS‐c treatment significantly lowered fasted blood glucose levels (*P *= 0.0279; Figure 1a) but did not alter non‐fasted blood glucose levels (*P* = 0.2312; Figure 1b). No difference in glucose concentration levels was detected between control and MOTS‐c treatment groups (*P* = 0.2700).

Circulating markers of inflammation included lactate dehydrogenase (LDH) and C‐reactive protein (CRP). LDH levels were not significantly different between groups. CRP levels, although not significantly increased in T2DM rats compared to controls (*P *= 0.5197), were significantly lower in the T2DM rats treated with MOTS‐c (*P *= 0.0287; Figure 1d).

Markers of general cardiac health included low‐density lipoprotein (LDL), high‐density lipoprotein (HDL), triglycerides and the LDL to HDL ratio. Compared to the control group, LDL levels were significantly higher in the T2DM group (*P* = 0.0032) and in MOTS‐c treatment (*P* < 0.0001), but no difference was found in the latter groups (*P* = 0.1454; Figure 1e). HDL and triglyceride levels did not differ significantly between groups (Figure 1f,h). Interestingly, although the ratio of LDL to HDL was not significantly different in T2DM rats compared with controls (*P* = 0.1551), it was higher in T2DM rats treated with MOTS‐c (*P* = 0.0424; Figure 1g).

Markers of liver function included albumin, alanine transaminase (ALT) and aspartate aminotransferase (AST). Albumin levels were unchanged between groups (Figure 1i). ALT was higher in T2DM rats than in controls (*P* = 0.0054), but remained unchanged with MOTS‐c treatment (*P *= 0.3989; Figure 1j). Conversely, AST was unchanged between controls and T2DM rats (*P *= 0.3983) but was significantly lower with MOTS‐c treatment (*P *= 0.0365; Figure 1k)

Kidney function markers included creatinine, urea, uric acid, sodium and chloride ions. Creatinine, urea, uric acid and sodium ion levels all showed no significant differences between groups (Figure 1i–o). Chloride ion levels were significantly higher in the T2DM rats compared to controls (*P *= 0.0022), but no difference between T2DM and MOTS‐c treated rats was detected (*P *= 0.9299; Figure 1p).

**FIGURE 2:**
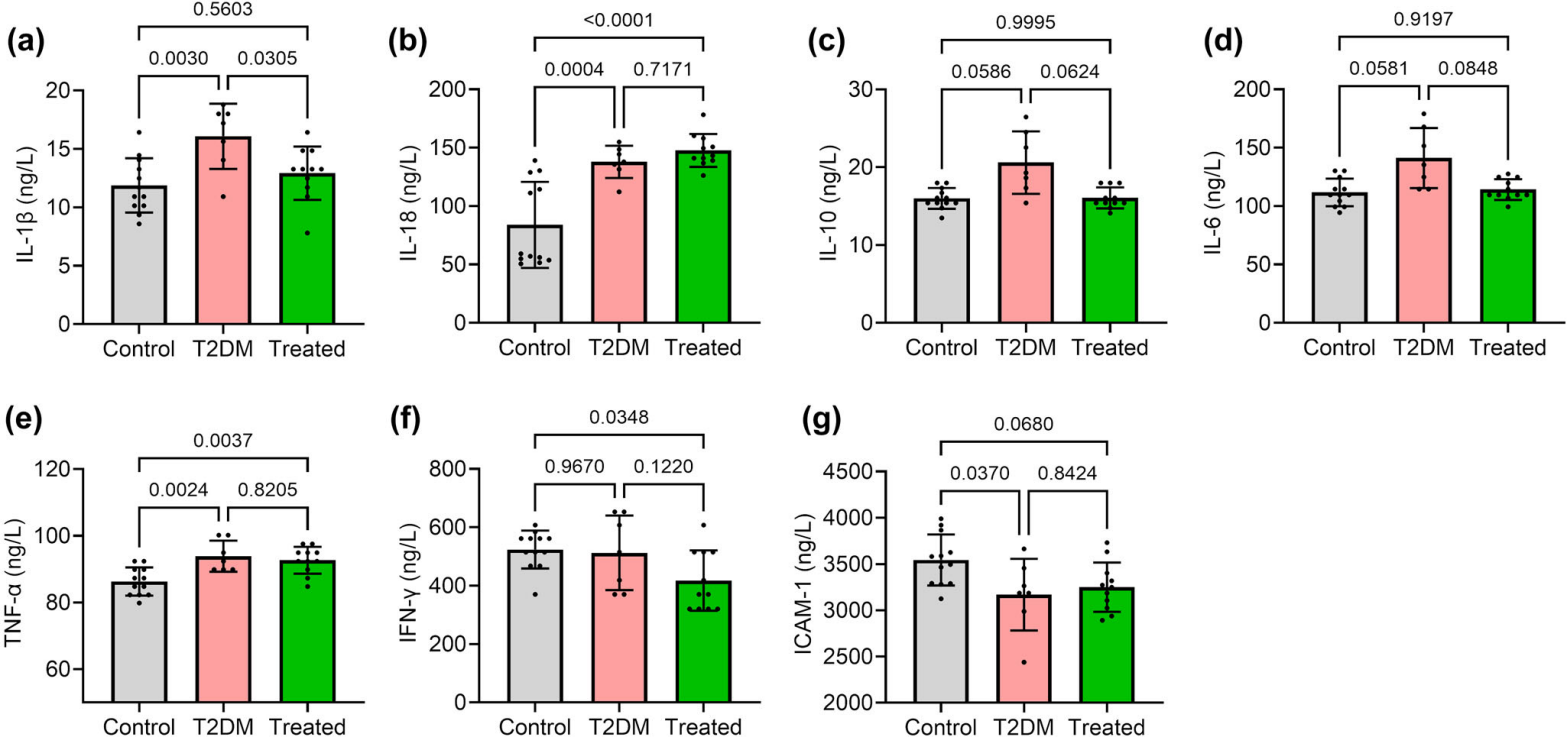
Inflammatory cytokines in the T2DM rat model and effect of MOTS‐c treatment. Inflammatory cytokines in the plasma from control rats, type 2 diabetes mellitus (T2DM) rats and T2DM rats treated with mitochondrial open reading frame of the 12S rRNA type‐C (MOTS‐c) were detected using the Luminex® assay. Data are means ± SD. Control (*n* = 12), T2DM (*n* = 7), Treated (*n* = 11). Ordinary one‐way ANOVA with Dunnett's multiple comparison of all groups to the T2DM group; Brown–Forsythe ANOVA with Dunnett's T3 multiple comparison of all groups to the T2DM group.

Inflammatory cytokines IL‐1β, IL‐18, IL‐10, IL‐6, TNF‐α, IFN‐γ and ICAM‐1 were quantified in blood samples using a Luminex® multiplex assay (Figure 2). Compared to controls, the levels of inflammasome‐derived inflammatory cytokines, IL‐1β (*P *= 0.0030; Figure 2a) and IL‐18 (*P *= 0.0004; Figure 2b), were significantly higher in the T2DM group compared with the control group, but only IL‐1β was significantly lowered with MOTS‐c treatment (*P *= 0.0305). IL‐10, an anti‐inflammatory cytokine produced to regulate inflammation, was not different in the T2DM group compared to controls (*P *= 0.0586) and to MOTS‐c treatment (*P *= 0.0624; Figure 2c). The pro‐inflammatory cytokine IL‐6 was not different between the groups (*P* > 0.0581; Figure 2d). Compared with the control group, the T2DM group showed significantly higher levels of the pro‐inflammatory cytokine TNF‐α (*P* = 0.0024), which remained unchanged in the MOTS‐c treatment group (*P* = 0.8205; Figure 2e). The cytokine IFN‐γ, crucial for immune cell activation and modulation, showed no significant differences between groups, although there was a trend towards reduction with MOTS‐c that did not reach statistical significance (*P* = 0.1220; Figure 2f). ICAM‐1, involved in immune cell migration, was the only marker to show a reduction in the T2DM model compared to controls (*P* = 0.0370), and this reduction remained unchanged with MOTS‐c treatment (*P* = 0.8424; Figure 2g).

**FIGURE 3:**
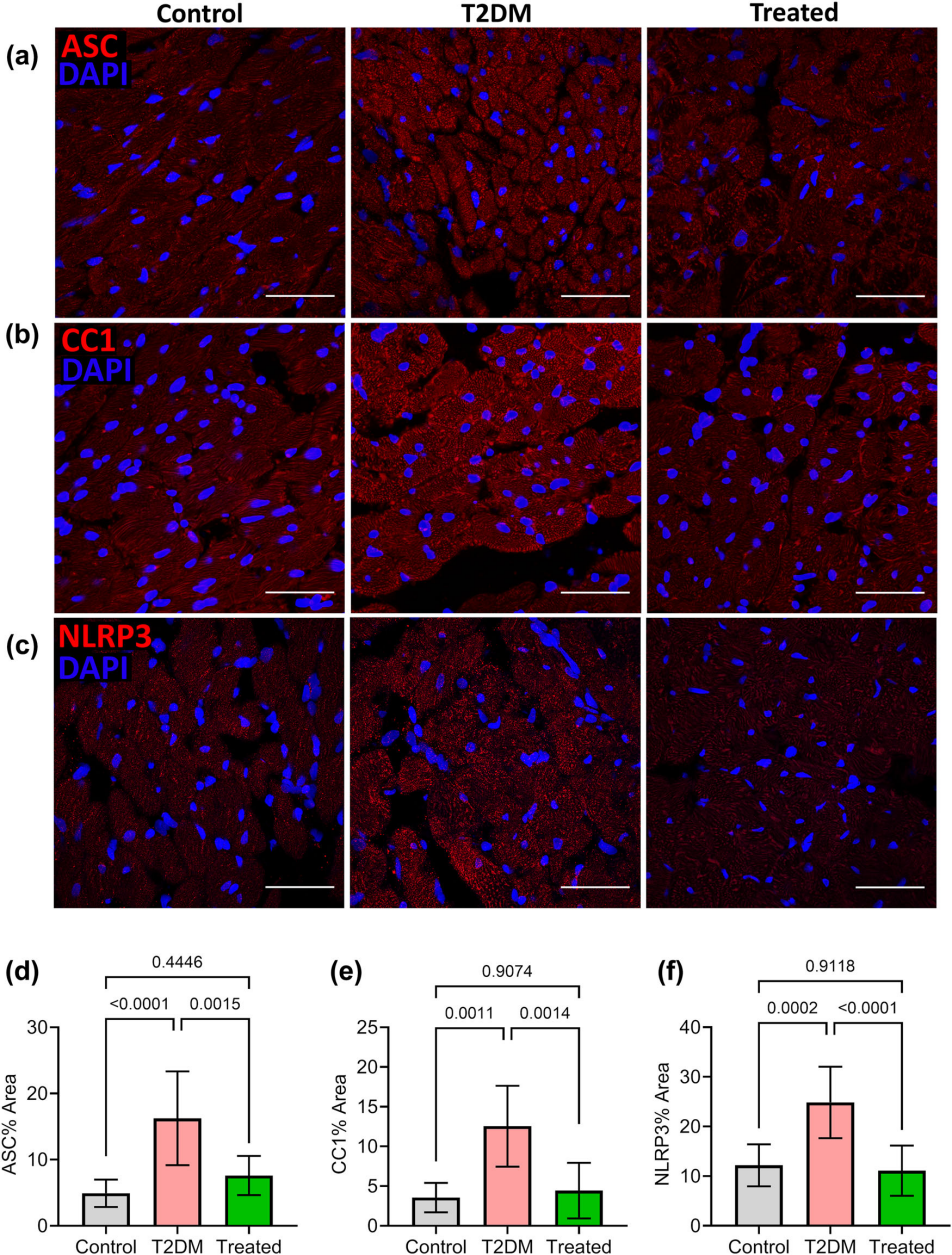
MOTS‐c lowers elevated inflammasome markers in the T2DM heart. Representative images of (a) apoptosis‐associated speck‐like protein containing a caspase activation and recruitment domain (ASC); (b) cleaved caspase‐1 (CC1); and (c) nucleotide‐binding oligomerisation domain, leucine‐rich repeat and pyrin domain‐containing receptor 3 (NLRP3) in left ventricle heart tissue from control rats (*n* = 6), type 2 diabetes mellitus rats (T2DM) (*n* = 6), and T2DM rats treated with mitochondrial open reading frame of the 12S rRNA type‐C (MOTS‐c) (*n* = 6). Scale bars: 50 µm. Quantification of fluorescence signal revealed significantly higher ASC (d), CC1 (e), and NLRP3 (f) percentage area coverage in the T2DM compared to controls that was significantly lowered with MOTS‐c treatment. Data are means ± SD; Brown–Forsythe ANOVA with Dunnet's T3 multiple comparison of all groups to the T2DM group; ordinary one‐way ANOVA with Dunnett's multiple comparison of all groups to the T2DM group.

Left ventricle heart tissue showed similar trends in all three markers of the NLRP3 inflammasome: ASC, CC1 and NLRP3 (Figure 3a–c). Compared to controls, the T2DM group demonstrated significantly higher percentage area covered by ASC (+11.3%, *P *< 0.0001), CC1 (+8.99%, *P *= 0.0011) and NLRP3 (+12.66%, *P *= 0.0002) fluorescence signal (Figure 3d–f). T2DM rats treated with MOTS‐c showed significantly lower ASC (−8.64%, *P *= 0.0125), CC1 (−8.107%, *P *= 0.0014) and NLRP3 (−13.75%, *P *< 0.0001) percentage area coverage (Figure 3d–f). No differences in ASC, CC1 or NLRP3 were found between the control and MOTS‐c groups (*P* > 0.4446).

**FIGURE 4:**
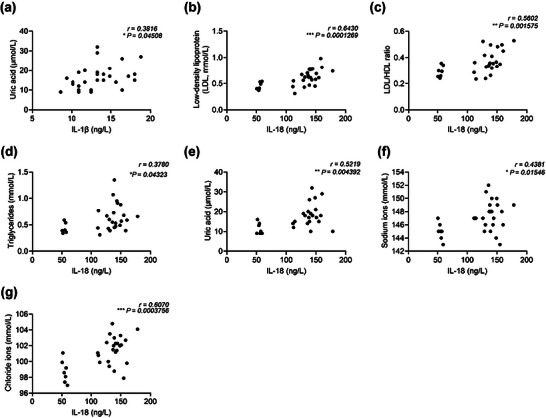
Significant correlations between Circulating markers and inflammasome cytokines. Significant correlations were found between the inflammasome‐derived inflammatory cytokines IL‐1β and IL‐18 with circulating markers of general health measured in plasma. *r* is Pearson's correlation coefficient. *P*‐values are two‐tailed. Control (*n* = 6), T2DM (*n* = 6), Treated (*n* = 6).

Correlations between the inflammasome‐derived cytokines IL‐1β and IL‐18 and circulating markers of general health were investigated. IL‐1β demonstrated a weak positive correlation with uric acid (*P *= 0.04508) (Figure 4a), but no significant relationships with any other markers. IL‐18 showed strong positive relationships with circulating LDL (*P *= 0.0001; Figure 4b) and chloride ions (*P *= 0.0004; Figure 4g), a weak positive relationship with triglycerides (*P *= 0.0432; Figure 4d), as well as moderate positive relationships with LDL/HDL ratio (*P *= 0.0016; Figure 4c), uric acid (*P *= 0.0044; Figure 4e) and sodium ions (*P *= 0.0155; Figure 4f).

## 4 DISCUSSION

**FIGURE 5:**
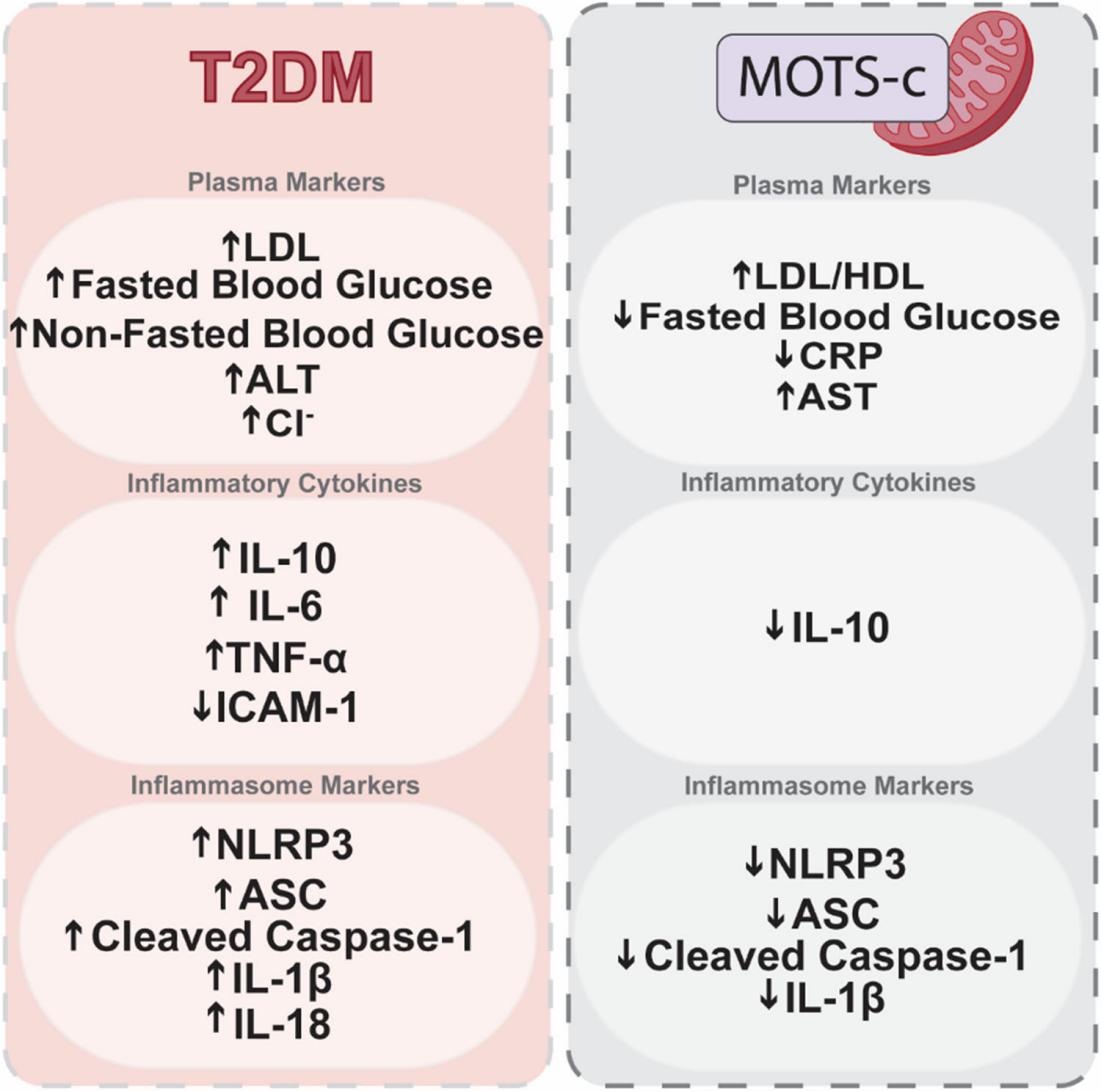
The effect of MOTS‐c treatment on plasma markers of general health, inflammatory cytokines, and cardiac inflammasome presence in T2DM. Summary of the changes to plasma markers of general health and circulating cytokines, and in the presence of cardiac inflammasome protein markers in the type 2 diabetes mellitus (T2DM) rat model and the effect of MOTS‐c treatment. The T2DM model showed elevated low‐density lipoprotein (LDL), fasted blood glucose, non‐fasted blood glucose, alanine transaminase (ALT), chloride ions, interleukin (IL)‐10, IL‐6, tumour necrosis factor α (TNF‐α), nucleotide‐binding oligomerisation domain, leucine‐rich repeat and pyrin domain‐containing receptor 3 (NLRP3), apoptosis‐associated speck‐like protein containing a caspase activation and recruitment domain (ASC), cleaved caspase‐1 (CC1), IL‐1β, IL‐18 and decreased intercellular adhesion molecule‐1 (ICAM‐1). MOTS‐c treatment significantly reduced fasted blood glucose, C‐reactive protein (CRP), IL‐10, NLRP3 ASC, CC1, IL‐1β, and increased LDL/HDL ratio and aspartate aminotransferase (AST).

This study provides a comprehensive analysis of the regulatory effects of MOTS‐c on inflammation and metabolism in the context of T2DM, with a particular focus on the NLRP3 inflammasome pathway (Figure 5). Our findings reveal that T2DM significantly increases in the presence of NLRP3, ASC and CC1, indicating inflammasome priming and activation. MOTS‐c treatment substantially reduces the presence of these inflammasome markers in diabetic heart tissue, consistent with its known activation of AMPK, which inhibits nuclear factor κB (NF‐κB)‐mediated transcription of NLRP3 and pro‐IL‐1β (Wan et al., 2023). This suppression is reflected in the plasma cytokine profile, where MOTS‐c lowers IL‐1β and IL‐10, but not IL‐18, IL‐6 or TNF‐α, indicating selective modulation of inflammatory signalling.

The selective reduction of IL‐1β but not IL‐18 by MOTS‐c treatment, despite both cytokines being elevated in the T2DM model, may reflect differences in their regulation and endogenous inhibitory mechanisms. Unlike IL‐1β, pro‐IL‐18 is constitutively expressed in many cell types, suggesting that its levels may be less sensitive to acute inflammatory modulation (Puren et al., 1999). Additionally, the pathways that regulate free cytokine availability differ markedly between IL‐1β and IL‐18. IL‐18 is neutralised by IL‐18 binding protein (IL‐18BP), which binds free IL‐18 and limits its bioactivity (Wang, 2024). In inflammatory states, IL‐18BP levels often rise in parallel with IL‐18, but this increase may be insufficient to fully sequester the cytokine, resulting in elevated free IL‐18 (Wang, 2024). Under MOTS‐c treatment, a concurrent reduction in both IL‐18 and IL‐18BP could yield no net change in measurable IL‐18. In contrast, IL‐1β is primarily regulated by receptor‐level antagonism. The endogenous IL‐1 receptor antagonist, also known as anakinra, competes with IL‐1β for type I IL‐1 receptor binding, while the type II receptor acts as a decoy lacking signalling capacity (Dinarello, 2011). These mechanisms do not alter the concentration of free IL‐1β, making it a more direct biomarker of inflammasome activity and a clearer target for assessing the effects of MOTS‐c.

Cytokine correlation analysis further elucidates the mechanistic specificity of MOTS‐c. IL‐18 and IL‐1β levels correlate positively with plasma uric acid, and IL‐18 also shows a positive correlation with LDL. Both uric acid and LDL are known to activate NLRP3 via mitochondrial ROS and lysosomal destabilisation (Wang et al., 2022; Wen et al., 2021). Additionally, elevated uric acid levels can indicate reduced kidney function, and hyperuricaemia can predict the development of kidney disease (Johnson et al., 2013). The only kidney function‐related marker that showed elevation in the T2DM group was chloride ions. In contrast, other markers, including urea, uric acid, creatinine and sodium ions, remained unchanged compared to the controls, suggesting that the T2DM group had not developed kidney dysfunction at the time point investigated. However, the positive relationships between uric acid, chloride ions and sodium ions with IL‐18 suggest a link between inflammasome activity and kidney function even before detectable pathology, which could be important in the development of diabetic kidney dysfunction.

Metabolic profiling revealed further complexity. T2DM increased both fasted and non‐fasted blood glucose levels, whereas MOTS‐c selectively reduced fasted blood glucose levels, suggesting an effect on basal glucose metabolism rather than postprandial regulation. This finding aligns with the role of MOTS‐c in enhancing insulin sensitivity and mitochondrial glucose metabolism (Lee et al., 2015). Interestingly, fasted blood glucose showed no relationship with IL‐1β or IL‐18, suggesting that MOTS‐c affects glucose metabolism and the NLRP3 inflammasome through distinct pathways. Plasma LDL levels were elevated in T2DM but were not reduced by MOTS‐c. Instead, the LDL/HDL ratio was elevated in T2DM treated with MOTS‐c, a finding that is somewhat consistent with recent findings showing elevated plasma MOTS‐c in obesity, reduced MOTS‐c in established diabetes, and positive correlations between MOTS‐c and total cholesterol and LDL‐c in individuals with obesity (Zhou et al., 2024), reflecting the early stage of diabetes in our rat model.

In addition, AST was higher in the MOTS‐c group. Previous studies report MOTS‐c to exert a positive impact on liver health (Chen et al., 2025; Lin et al., 2024) and to reduce LDL levels (Tang et al., 2023) and hepatic lipid accumulation (Lee et al., 2015). The therapeutic effects of MOTS‐c are highly dose‐dependent, with no optimal dosage established across diseases, potentially explaining discrepancies between studies (Fang et al., 2025). While lower doses (0.5–5 mg/kg) are typically administered over longer durations, higher doses (10–15 mg/kg) yield faster effects but may pose greater side effects, including potential liver function impairment, warranting further research into ideal dosing strategies. As IL‐18 showed significant positive correlations with LDL levels, triglyceride levels and the LDL/HDL ratio, and elevated IL‐18 was not affected by MOTS‐c, perhaps the regulation of this cytokine, alongside MOTS‐c's regulation of IL‐1β, may provide a broader approach capable of regulating LDL levels and liver function.

Interestingly, MOTS‐c reduced CRP levels, despite CRP not being elevated in T2DM, suggesting a broader anti‐inflammatory effect independent of acute‐phase reactants. CRP is produced by the liver in response to inflammatory cytokines IL‐6 and IL‐1β (Kramer et al., 2008). CRP is also known to activate NLRP3 via FcγR/NF‐κB signalling and promote LDL transcytosis (Bian et al., 2019), placing it at a critical intersection of metabolic and inflammatory pathways. The broad anti‐inflammatory effect of MOTS‐c is further supported by the regulatory cytokine IL‐10. IL‐10 is expressed by stimulated inflammatory cells to prevent hyper‐inflammation, maintain cell homeostasis and moderate apoptosis (Saraiva & O'garra, 2010). Elevated IL‐10 in T2DM and subsequent reduction with MOTS‐c may indicate lower overall inflammation, requiring less endogenous IL‐10 intervention.

Several limitations should be acknowledged. The selective reduction in fasted but not non‐fasted glucose points to a need for more detailed metabolic flux analysis. The study also did not address cell‐type‐specific responses, which may be critical given systemic circulation and the mitochondrial origin of MOTS‐c. Variability in MOTS‐c dosing and treatment duration across studies limits direct comparisons, highlighting the need for a standardised therapeutic protocol. We note that MOTS‐c effects are dose‐dependent, and the optimal doses remain undefined across disease contexts. Determining the optimal balance between dose, duration, efficacy and safety of MOTS‐c treatment is urgently required in future investigations. Additionally, biological responses to both disease contexts and treatments can be influenced by strain, sex and age. Therefore, the exclusive use of male Wistar rats may limit the applicability of our findings to females or to genetically diverse populations. Lastly, the study would be more insightful if it included echocardiographic measurements or an *ex vivo* working heart to assess cardiac contractile function.

Future studies should investigate the temporal dynamics and tissue‐specific distribution of MOTS‐c, including its uptake and signalling in immune, hepatic and cardiac cells. Combining MOTS‐c with redox modulators or lipid‐lowering agents may enhance its therapeutic efficacy. Transcriptomic and metabolomic profiling of affected tissues could uncover downstream gene networks and metabolic pathways regulated by MOTS‐c. Additionally, exploring the interaction between MOTS‐c and other mitochondrial peptides may reveal synergistic mechanisms. Clinical trials assessing MOTS‐c analogues in metabolic and inflammatory diseases are warranted to translate these findings into therapeutic applications.

### 4.1 Conclusion

These findings demonstrate that MOTS‐c effectively suppresses systemic and cardiac NLRP3 inflammasome activation in a diabetic rat model, highlighting its potential as a therapeutic agent for mitigating inflammation‐driven cardiomyopathy and related complications in type 2 diabetes.

## AUTHOR CONTRIBUTIONS

Toan Pham and Odunayo O. Mugisho conceived and designed the study. All authors performed the experiments, analysed the data and co‐wrote the manuscript. All authors have read and approved the final version of this manuscript and agree to be accountable for all aspects of the work in ensuring that questions related to the accuracy or integrity of any part of the work are appropriately investigated and resolved. All persons designated as authors qualify for authorship, and all those who qualify for authorship are listed.

## CONFLICT OF INTEREST

The authors declare that the research was conducted in the absence of any commercial or financial relationships that could be construed as a potential conflict of interest.

## Data Availability

The data that support the findings of this study will be made available from the corresponding author upon request.
